# Strategies for the development of stimuli-responsive small molecule prodrugs for cancer treatment

**DOI:** 10.3389/fphar.2024.1434137

**Published:** 2024-07-31

**Authors:** Yuxuan Tu, Jianbao Gong, Jing Mou, Hongfei Jiang, Haibo Zhao, Jiake Gao

**Affiliations:** ^1^ The Afffliated Hospital of Qingdao University, Qingdao University, Qingdao, China; ^2^ Qingdao Hospital, University of Health and Rehabilitation Sciences, Qingdao Municipal Hospital, Qingdao, China; ^3^ Department of Neonatology, Qingdao Women and Children’s Hospital, Qingdao University, Qingdao, Shandong, China

**Keywords:** prodrug, anti-tumor drug, cancer treatment, drug activation, stimuli-responsive, tumor micro-environment

## Abstract

Approved anticancer drugs typically face challenges due to their narrow therapeutic window, primarily because of high systemic toxicity and limited selectivity for tumors. Prodrugs are initially inactive drug molecules designed to undergo specific chemical modifications. These modifications render the drugs inactive until they encounter specific conditions or biomarkers *in vivo*, at which point they are converted into active drug molecules. This thoughtful design significantly improves the efficacy of anticancer drug delivery by enhancing tumor specificity and minimizing off-target effects. Recent advancements in prodrug design have focused on integrating these strategies with delivery systems like liposomes, micelles, and polymerosomes to further improve targeting and reduce side effects. This review outlines strategies for designing stimuli-responsive small molecule prodrugs focused on cancer treatment, emphasizing their chemical structures and the mechanisms controlling drug release. By providing a comprehensive overview, we aim to highlight the potential of these innovative approaches to revolutionize cancer therapy.

## 1 Introduction

Cancer is a disease caused by the abnormal proliferation of cells, leading to uncontrollable cell growth and the formation of primary solid tumors which can invade adjacent and distant sites, a process known as cancer metastasis (Fares et al., 2020; Bian et al., 2022). Metastasis is a major factor in the failure of cancer therapy, resulting in a mortality rate exceeding 90% among cancer patients (Steeg, 2006). In 2023, the American Cancer Society estimated that there will be approximately 1.96 million new cancer cases and 6,09,820 cancer-related deaths in the United States alone (Siegel et al., 2023). Consequently, researchers are focusing on developing new and effective cancer treatment options, with significant efforts dedicated to exploring potent chemotherapeutics.

**FIGURE 1:**
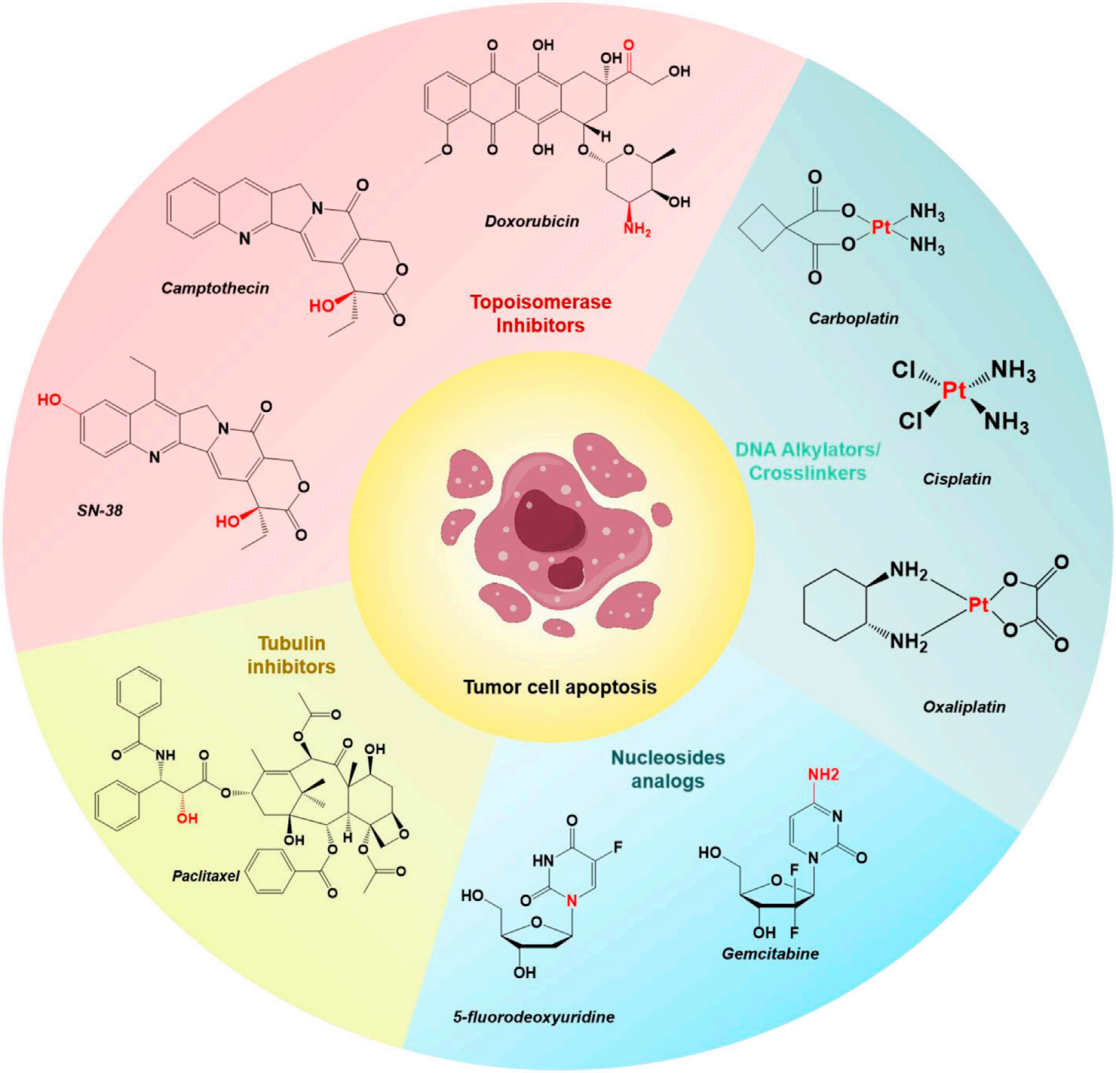
Chemical structures of select examples of FDA-approved chemotherapeutics, and their common modifiable moieties for synthesizing prodrugs.

The U.S. Food and Drug Administration (FDA) has approved various small-molecule drugs for cancer chemotherapy, including doxorubicin (DOX), camptothecin (CPT) and its derivative SN-38, gemcitabine (GEM), paclitaxel (PTX), 5-fluorodeoxyuridine (FDU), cisplatin, carboplatin, and oxaliplatin (Figure 1) (Liu et al., 2023; Yang et al., 2023; Zhang et al., 2023; Zhao et al., 2023). These chemotherapeutic agents act through various mechanisms: DOX and CPT inhibit topoisomerases, preventing DNA replication and transcription; PTX stabilizes microtubules, inhibiting tubulin depolymerization and arresting cell division; GEM and FDU interfere with DNA synthesis by incorporating into the DNA strand; and cisplatin, carboplatin, and oxaliplatin form DNA crosslinks, disrupting DNA structure and function, ultimately leading to cell apoptosis and tumor growth inhibition (Nitiss, 2009; Shewach and Kuchta, 2009; Huang and Zhou, 2021; Ebenezer et al., 2022). However, these drugs often lack specificity for targeting tumor cells over normal healthy cells, leading to severe side effects.

Therefore, there is a growing focus on developing novel and potent chemotherapeutics with new mechanisms of action to more effectively target and treat cancers. The construction of effective chemotherapeutics with improved aqueous solubility, enhanced cell uptake, and specific targeting of tumors is highly valued in the field.

In recent years, the development of prodrug-based strategies for cancer therapy has gained considerable attention (Vedagopuram and Bajaj, 2019; Sreekanth et al., 2021; Couvreur et al., 2023). Through selected structure modifications, anticancer prodrugs are inactive chemotherapeutic agents that possess little or no pharmacological activity within non-target tissues. Subsequently, in the presence of a tumor-specific biomarker, the “modified” prodrugs can be converted into active chemotherapeutics to produce a therapeutic effect at the target locations. The enhanced specificity of anticancer prodrugs minimizes the off-target toxicity of the drug and enhances the therapeutic efficacy. Several examples of prodrugs have been reported, which have high stability, high tumor specificity, and high maximum tolerated doses, while off-target toxicities are minimal (Amly and Karaman, 2016; Rautio et al., 2018; Souza et al., 2019). Moreover, the aqueous solubility and cell permeability of some prodrugs have also been improved through purposeful designs (Rautio et al., 2008; Sanches and Ferreira, 2019). The FDA has respectively approved the anticancer prodrugs Zytiga (for metastatic castration-resistant prostate cancer) in 2011 and Ninlaro (for multiple myeloma) in 2015 (Rehman and Rosenberg, 2012; Shirley, 2016).

**FIGURE 2:**
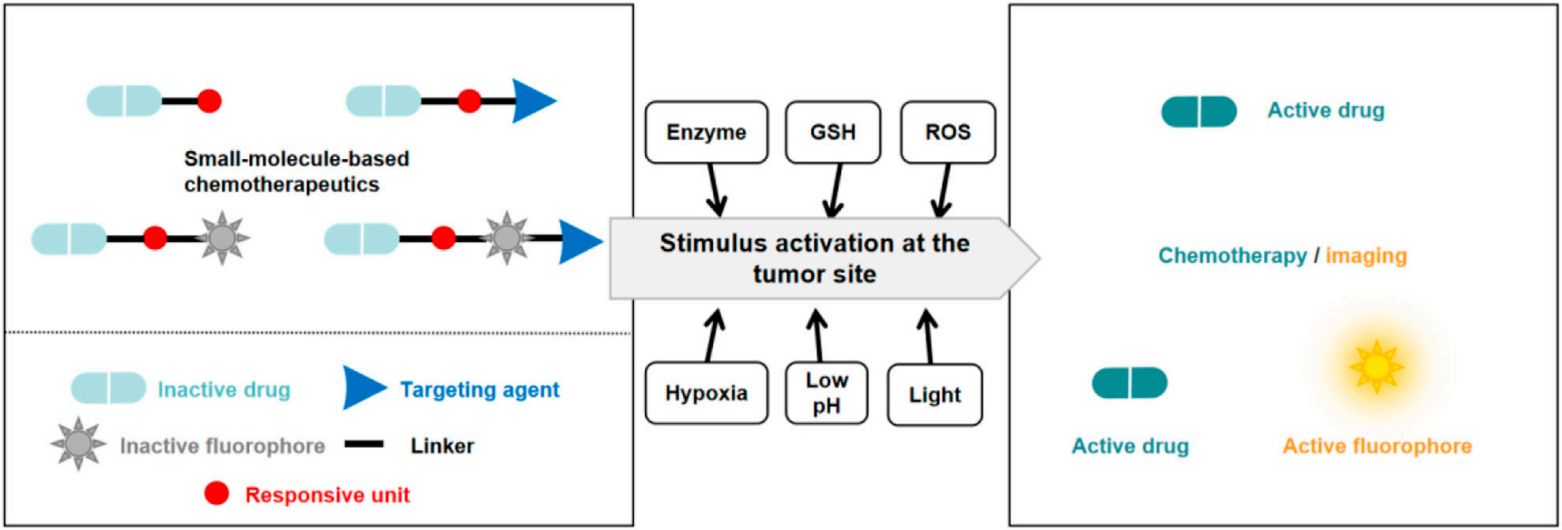
Basic schematic diagram of the various design strategies used to create small molecule prodrugs.

Small molecule prodrugs have small molecular weights and have attracted considerable research interest during the development of anticancer prodrugs. Early prodrug examples were designed to enhance solubility, cell permeability, and chemical stability. In recent years, more attention has been focused on overcoming the toxicity issues surrounding chemotherapeutics, coupled with the aim of adding some additional features such as diagnosis and drug release tracking properties (Meng et al., 2016; Walther et al., 2017; Goetz and Schork, 2018). These design strategies are expected to achieve better therapeutic effects. In order to reach the targeted goals, the development of anticancer prodrugs typically requires synthetic modification of a chemotherapeutic agent to mask its cytotoxicity. Modification methods include the introduction of biomarker-responsive chemical groups or light-responsive groups (Figure 2). Specific biomarkers are present within the tumor microenvironment (TME), which consists of blood vessels, endothelial cells, immune cells, rapidly proliferating cancer cells, fibroblasts, and an extracellular matrix (Wu and Dai, 2017; Li et al., 2022; Chen B.-Q. et al., 2023). To sustain this microenvironment, several physiological processes are regulated, resulting in specific traits of the TME. For example, the TME has a lower pH (ranging from 6.5 to 7.2) compared to that of normal tissues (with a pH around 7.4) owing to the Warburg effect (Liberti and Locasale, 2016), and it is hypoxic due to the insufficient blood supply to tumor tissue and the increased oxygen consumption by cancer cells (Chen Z. et al., 2023). The levels of reactive oxygen species (ROS) and enzymes are also elevated in the TME. In addition, intracellular biomarkers such as glutathione (GSH), β-galactosidase (β-Gal), and γ-glutamyltranspeptidase (GGT) are upregulated in cancers. These traits can be utilized for the design of anticancer small molecule prodrugs (Han et al., 2023). In this review, we discuss recently reported small molecule prodrug strategies for cancer treatment, focusing on small molecule prodrugs that can be activated by specific biomarkers in tumor issues or by light.

## 2 Small molecule prodrugs

### 2.1 Enzyme-responsive small molecule prodrugs

Enzyme-responsive anticancer small molecule prodrugs are modified drugs that can release functional moieties with the catalysis of enzymes. Some enzymes are typically significantly upregulated or specifically expressed in tumors, such as carboxylesterases (Li et al., 2017; Dai et al., 2022), β-galactosidase (Asanuma et al., 2015), β-glucuronidase (Cheng et al., 2021), NAD(P)H: quinone oxidoreductase 1 (NQO1) (Wu et al., 2018), and prostate-specific antigen (PSA) (Jiang and Hu, 2013). The elevated enzymatic activities promote pathological processes including tumor cell angiogenesis, cell invasion, and metastasis (Baig et al., 2019). Based on the types of cancer and the anticancer drug mechanism, various combinations of enzyme-specific linkers and anticancer drugs constitute the diverse structure of prodrugs. Table 1 summarizes some target enzymes for the prodrugs.

**TABLE 1:** Summary of some enzyme-responsive small molecule prodrugs.

Enzymes	Substrates	Antitumor drugs	*In vitro* models	*Ex vivo* */* *in vivo* models	Ref
Carboxylesterase	Ester, thioester, carbamate, and amide bonds	Gemcitabine	Huh7, HepG2, HEK293, MCF-7, BxPC3	—	Stephenson et al. (2021)
		DOX	MCF-7, HepG2, HT-29, HeLa, NIH3T3	—	Bliman et al. (2018); Sharma et al. (2019)
		Docetaxel	22Rv1, PC-3	22Rv1 tumor-bearing mice	Machulkin et al. (2022)
		5-Fluorouracil	HeLa, MG36	mice	Ouyang et al. (2011)
β-Galactosidase	Glycosidic bond	DOX	HT-29, HeLa	HT-29 tumor-bearing mice	Sharma et al. (2018a)
		MMAE	KB, HeLa	KB tumor-bearing mice	Legigan et al. (2012)
β-Glucuronidase	β-D-Glucuronic acid residues	SN-38	A594, HCT116, MCF-7, HepG2	HCT116 tumor-bearing mice	Huang et al. (2021)
		MMAE	KB, A549, MDA-MB-231, MIA PaCa2	KB tumor-bearing mice	Renoux et al. (2017)
		CA-4	HeLa, MDA-MB-231, MCF-7, SKOV-3	MDA-MB-231 tumor-bearing mice	Peng et al. (2022)
NQO1	Quinones	5-Fluorouracil	A549, L02	A549 tumor-bearing mice	Zhang X. et al. (2018)
		CA-4	HepG2, A549	HepG2 tumor-bearing mice	Zhang et al. (2020)
		SN-38	A549, HeLa	A549 tumor-bearing mice	Shin et al. (2016)
PSA	-Arg-Ser-Ser-Tyr-Tyr Ser-Arg-	PTX	LNCaP	LNCaP tumor-bearing mice	Elsadek et al. (2010)
	-Hyp-Ala-Ser-Chg-Gln-	Phosphoramide mustard	LNCaP	—	Wu and Hu (2016)
	-His-Ser-Ser-Lys-Leu-Gln-	Emetine	LNCaP, 22Rv1	—	Akinboye et al. (2017)
Caspase-3	-Asp-Glu-Val-Asp-	DOX	SCC7, MDA-MB-231	SCC7 tumor-bearing mice	Chung et al. (2016)
		MMAE	HCC70, MDA-MB-231	HCC70/MDA-MB-231 tumor-bearing mice	Chung et al. (2019)
Alkaline phosphatase	Phosphate group	5-Fluorouracil, vorinostat	HeLa, HepG2	—	Tyagi et al. (2023)
Plasmin	D-Ala-Leu-Lys-	Si113	HepG2, Huh-7	HepG2 tumor-bearing mice	Rango et al. (2021)
Cathepsin B	Peptide	Gemcitabine	4T1	4T1 tumor-bearing mice	Zhang H. et al. (2018)
FAPα	N-terminal carbobenzoxy-glycyl-L-proline-4-(Z)-blocked dipeptides (Z-GP)	Gemcitabine	4T1, PC-3	4T1 tumor-bearing mice	Sun et al. (2019)
Tyrosinase	Phenol	Cyclometalated Pt (II) complex	A375	A375 tumor-bearing mice	Liu Y. et al. (2023)
γ-Glutamyltransferase	Glutamyl group	Kinesin spindle protein inhibitors	A549, H460	—	Fukai et al. (2021)

Carboxylesterase (CE) is essential for metabolizing xenobiotics, including drugs and toxins, and is regarded as a useful tumor biomarker for patient staging, which can catalytically hydrolyze several carboxyl esters and amides (Wang et al., 2018; Lucy and Hao-Jie, 2020). Therefore, several CE-responsive small molecule prodrugs have been developed for the treatment of cancer (Ouyang et al., 2011; Bliman et al., 2018; Sharma et al., 2019; Stephenson et al., 2021; Machulkin et al., 2022). These prodrugs have esterase-activable moieties to mask the original toxicity of antitumor drugs or to optimize their therapeutic efficacy.

**FIGURE 3:**
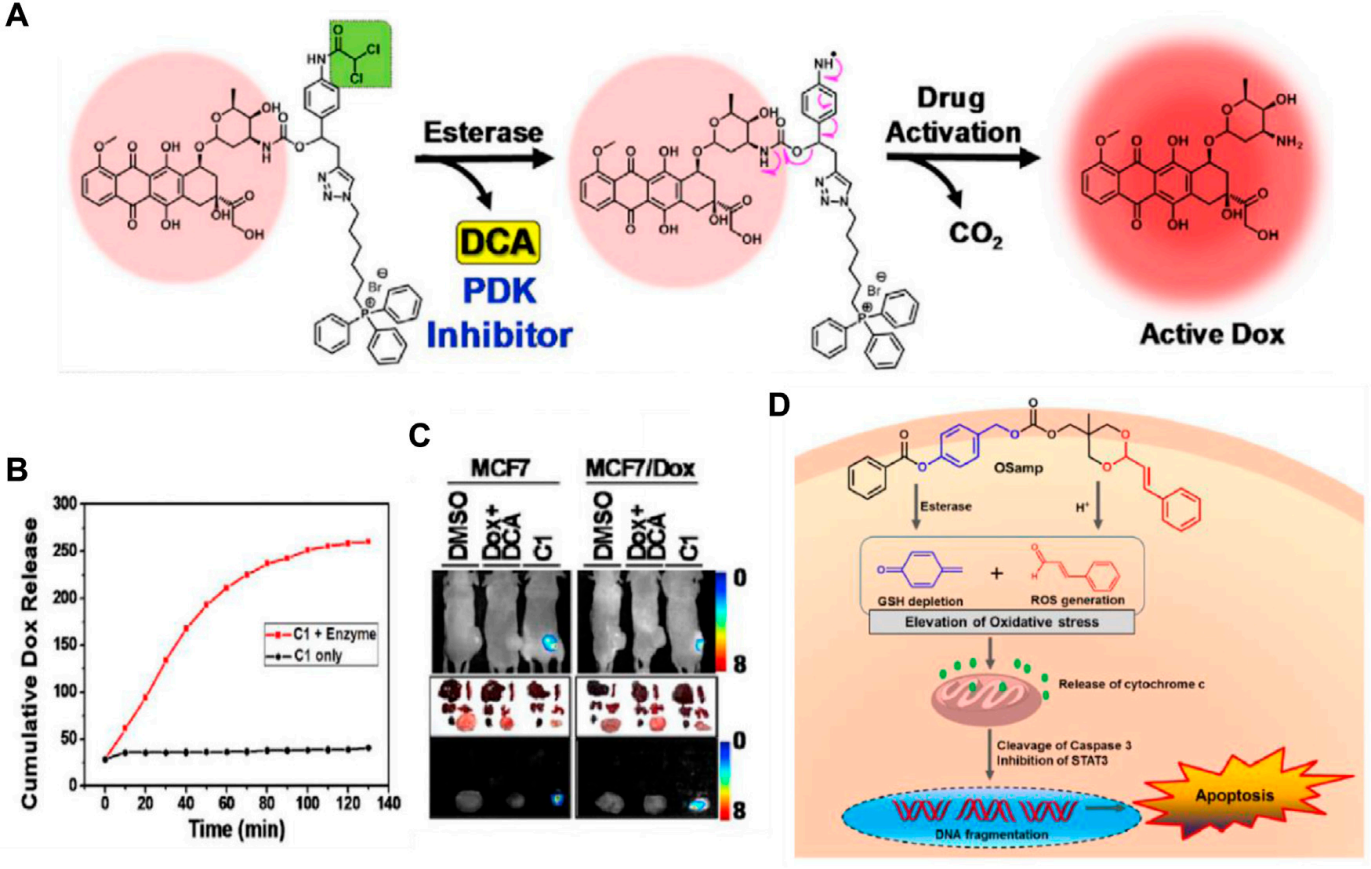
**(A)** The chemical structure and activation mechanism of C1 by carboxylesterase are depicted in the figure. **(B)** Time-dependent fluorescence enhancement seen upon incubation of C1 with carboxylesterase (1 U/mL) in PBS (37°C). The change in fluorescence intensity is directly related to active Dox release from C1. Adapted with modification from Ref. (Sharma et al., 2018b) ^©^ 2018 Elsevier. **(C)** Fluorescence images of mice with MCF7/Dox and MCF7 tumors and *ex vivo* images of dissected organs are provided as representations. **(D)** Chemical structure of OSamp, a hybrid anticancer prodrug activated by dual stimuli that amplifies oxidative stress. Adapted with modification from Ref. (Han et al., 2017) ^©^ 2017 American Chemical Society.

Chemotherapy is crucial for the treatment of cancer. However, drug resistance represents the primary barrier to the successful treatment effects of chemotherapy (Holohan et al., 2013). Tumors become resistant due to the rapid growth of small subpopulations of resistant cells that survive initial treatment. When resistance from one drug extends to other antitumor therapies, it leads to multidrug resistance (MDR) (Gottesman, 2002). This resistance often results from increased drug efflux mediated by ATP-binding cassette (ABC) transmembrane proteins, such as ABCG2 and MDR1 (p-glycoprotein), which are key players in normal physiology and major contributors to chemoresistance (Gottesman et al., 2002). Although development of various p-glycoprotein inhibitors is a feasible strategy to overcome cancer MDR, enormous efforts have been dedicated to exporting other innovative designs such as targeted small molecule prodrugs, to circumvent MDR mechanisms (Duan et al., 2023). Kim and his colleagues have developed a small molecule targeted prodrug, compound C1, capable of overcoming drug resistance at low doses for MDR cells. C1 is composed of a DOX moiety linked reversibly to a triphenylphosphonium (TPP) mitochondrial targeting group and a dichloroacetic acid (DCA) subunit (Sharma et al., 2018b). Firstly, C1 is targeted to the cancer mitochondria by the lipophilic TPP moiety and accumulates, then becomes activated by CE. The released DCA inhibits PDK (a key enzyme in the metabolism of tumor cells), shifting the cancer cells’ metabolism from aerobic glycolysis to oxidative phosphorylation, aiding in overcoming drug resistance. The free DOX generated through self-immolation in the mitochondria translocates to the nucleus over time, where it functions (Figure 3A). *In vitro* release test showed that C1 would likely prove stable under physiologic conditions, while exposure of C1 to carboxylesterase (1 U/mL) resulted in scission of the aniline-amide bond and release of Dox from the reactive intermediate (Figure 3B). C1 and formulations of DCA + DOX (a mixture of DCA and DOX) were injected into mice with MCF7 and DOX-resistant MCF7/DOX xenograft tumors. C1 injection reduced tumor volume by about 78% (MCF7) and 74% (DOX/MCF7), while combined DCA + DOX treatment led to 53% and 18% reductions, respectively. Fluorescence image analysis revealed that C1 accumulated at tumor sites to release the drug DOX compared to the equivalent concentration of DCA + DOX (Figure 3C). These findings suggest C1 overcomes the efflux mechanisms in DOX-resistant cells and significantly inhibits the growth of resistant cell line xenografts (DOX/MCF7). The combination of subcellular targeting and metabolic alteration through an enzyme-responsive linker offers a promising strategy for tackling chemoresistant tumors.

The Lee group designed a novel hybrid anticancer prodrug (OSamp) that amplifies oxidative stress by depleting GSH and generating ROS (Han et al., 2017). OSamp was designed with two complementary pharmacophores: a cinnamaldehyde for ROS generation through acid-triggered hydrolysis, and a quinine methide for GSH scavenging through esterase-catalyzed hydrolysis. This design results in a significant accumulation of ROS, leading to preferential cancer cell death (Figure 3D). In the mouse model of SW620 tumor xenografts, OSamp (2 mg/kg) significantly increased oxidative stress, inducing apoptotic cell death and reducing tumor growth without notable side effects. This study highlights a dual stimuli-activatable prodrug approach, potentially extendable to the development of hybrid prodrugs.

**FIGURE 4:**
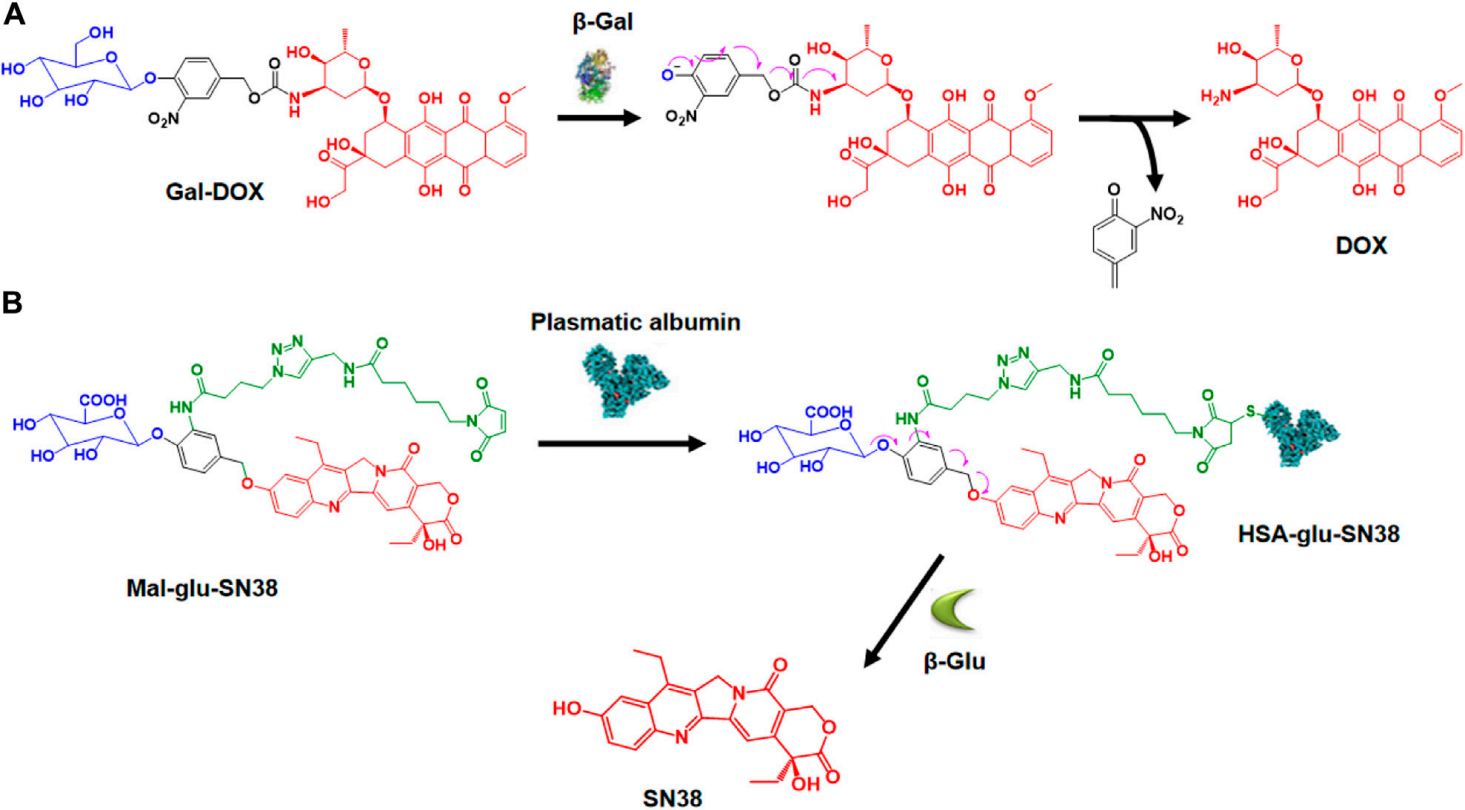
**(A)** Activation mechanism of Gal-DOX by β-galactosidase. Adapted with modification from Ref. (Sharma et al., 2018a) ^©^ 2018 Elsevier. **(B)** Creation of HSA-glu-SN38 and its mechanism of drug release. Adapted with modification from Ref. (Huang et al., 2021) ^©^ 2021 Elsevier.

β-Galactosidase (β-gal) is an enzyme that breaks down glycosidic bonds and hydrolyzes β-galactosides into monosaccharides. Overexpression of β-gal in tumors has been reported (Wagner et al., 2015), leading to the development of β-gal-responsive antitumor prodrugs. Kim and colleagues designed a theranostic system, Gal-DOX, which includes a galactose moiety and DOX connected by a self-immolative linker (Sharma et al., 2018a). After activation in cancer cells, the glycosidic bond is broken, releasing DOX for colon cancer therapy with a fluorescence turn-on response, enabling monitoring of drug localization and action site (Figure 4A). They observed that Gal-DOX’s toxicity did not significantly differ from free DOX in HT-29 cells but showed reduced cytotoxicity in HeLa cells with low β-gal expression. In HT-29 xenograft-bearing nude mice, Gal-DOX’s tumor-targeted accumulation was visualized by enhanced fluorescence up to 48 h, while free DOX cleared from the tumor site within 24 h. The treatment with Gal-DOX significantly retarded tumor growth, showing 53.1% tumor growth inhibition compared to 34.9% with free DOX treatment. Additionally, the Papot group developed a prodrug by combining a galactoside trigger with a folate receptor (FR) ligand, resulting in the tubulin inhibitor monomethyl auristatin E (MMAE) release (Legigan et al., 2012). This prodrug released free MMAE, inhibiting cancer cell division after specific internalization in KB cells through FR-mediated endocytosis and glycosidic bond degradation.

β-Glucuronidase (β-glu) hydrolyzes β-D-glucuronide compounds and is highly expressed in a broad range of tumors. β-glu is deemed to be associated with tumor invasion and metastasis (Tranoy-Opalinski et al., 2014). The strategy of employing β-glucuronidase-responsive prodrugs for the targeted delivery of small molecule drugs has been demonstrated to be feasible. Lu and colleagues designed an innovative β-glucuronidase-responsible albumin-binding prodrug, SN38-glu-Mal, to selectively deliver SN38 to tumor sites for maximum efficacy (Figure 4B) (Huang et al., 2021). In human HCT116, A549, HepG2, and MCF-7 cell lines, the prodrug’s toxicity was significantly lower than SN38. However, adding β-glucuronidase to the cell culture medium released SN38, causing increased cytotoxicity similar to SN38. SN38-glu-Mal (25 mg/kg) and irinotecan (15 mg/kg, metabolized to SN38) were injected into HCT116 tumor-bearing mice. SN38-glu-Mal accumulated more at the tumor site than irinotecan, with its concentration in tumor tissue 30 times higher after 48 h; The SN38-glu-Mal group showed lower circulating SN38 but increased tumor-site accumulation compared to irinotecan. Targeting the tumor microenvironment with β-glucuronidase-responsive prodrugs of MMAE and the tubulin inhibitor combretastatin A4 (CA-4) for efficient therapy has also been reported (Renoux et al., 2017; Peng et al., 2022).

**FIGURE 5:**
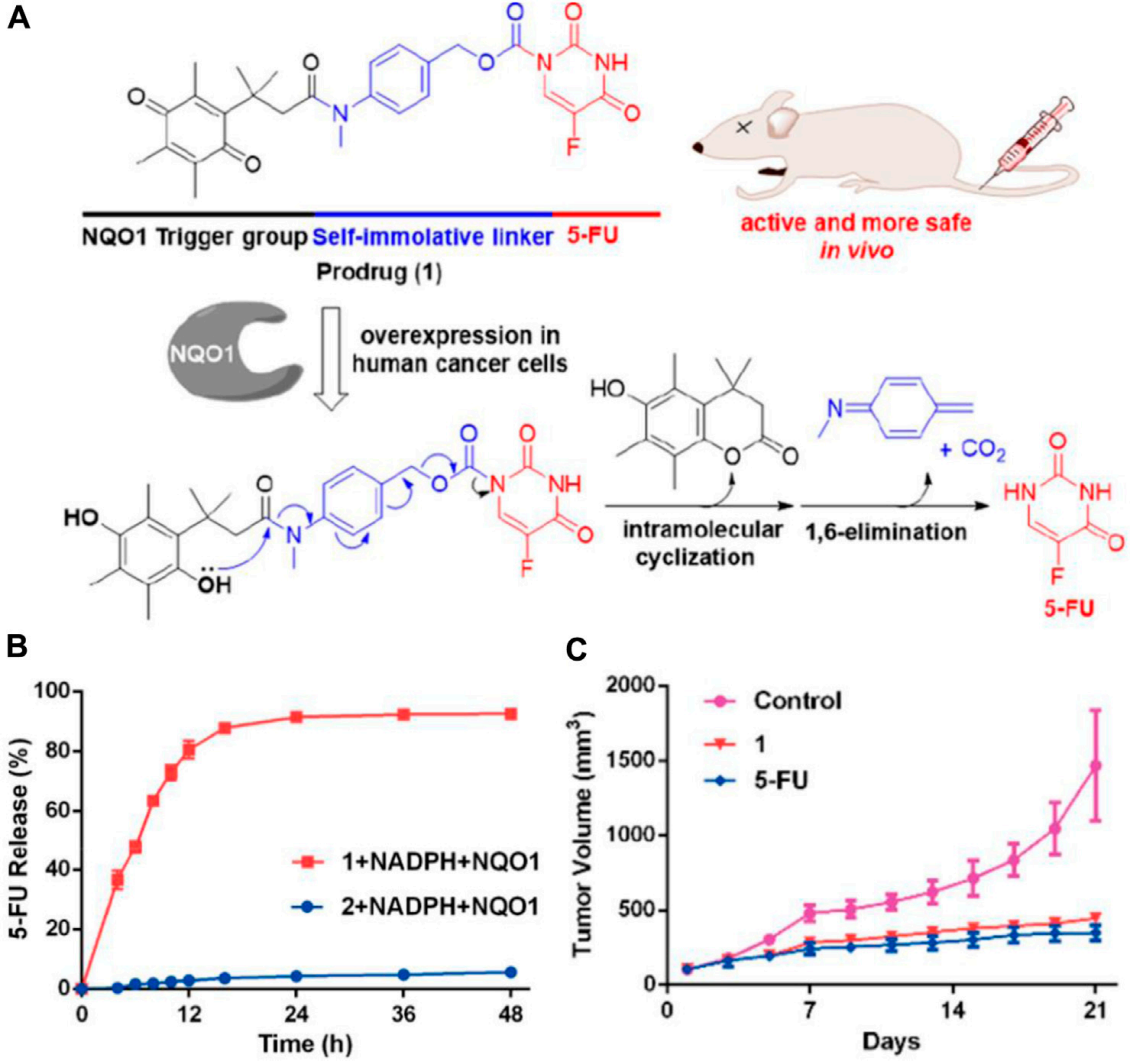
**(A)** Mechanism of the activation and chemical structure of the prodrug. **(B)** Percentage of 5-FU released from 1 (20 µM) and 2 (20 µM) over time in the presence of NADPH (100 µM) and NQO1 (14 μg/mL) in PBS. **(C)** Changes of tumor volumes (iv, 15 mg/kg). Adapted with modification from Ref. (Zhang et al., 2018) ^©^ 2018 American Chemical Society.

NQO1 is an enzyme that facilitates the reduction of quinones and is involved in detoxifying harmful mutagens or toxins. As NQO1 is highly expressed in many solid tumors, including those of the liver, lung, breast, and colon, it is used to activate quinone prodrugs. For instance, Zhang and coworkers synthesized a small molecule prodrug that includes an NQO1-responsive trigger group (Q3PA, quinone propionic acid), a self-immolative linkage, and the active drug 5-FU (Figure 5A) (Zhang et al., 2018). As NQO1-mediated reduction proceeds, Q_3_PA ester spontaneously forms lactone by intramolecular cyclization, which can lead to rapid release of the chemical moiety.

Compared to the compound generated through the condensation of 5-FU with Q3PA without the self-immolative linker, the prodrug steadily released 5-FU, and 5-FU’s concentration reached a plateau within 48 h (Figure 5B). The results from the study on the prodrug’s impact on tumor growth in A549 xenografts show that it is more effective at inhibiting tumor growth *in vivo* than the unmodified 5-FU compound (Figure 5C). Similarly, Wu’s group selected CA-4 as the drug to be connected with the NQO1-responsive trigger group via different self-immolative linkers and evaluated them biologically (Zhang et al., 2020). Furthermore, a cancer-targeting and NQO1-triggered theranostic prodrug containing SN-38 was developed by Kim and colleagues for a more effective cancer treatment (Shin et al., 2016).

Prostate-specific antigen (PSA) represents a serine protease specifically expressed in prostate tissue and carcinoma (Christensson et al., 1990). As it is overexpressed in the prostate tumor site but rapidly inactivated in systemic circulation due to binding to serum protease inhibitors like α2-macroglobulin (A2M) and α1-antichymotrypsin (ACT), PSA is considered an attractive target for small molecule prodrugs against prostate cancer (Lilja et al., 1991; Otto et al., 1998). Kratz’s group developed a PSA cleavable albumin-binding prodrug of PTX, which was approximately three times more cytotoxic than paclitaxel in PSA-positive LNCaP cells (Elsadek et al., 2010). Moreover, PSA activatable peptide prodrugs of phosphoramide mustard and emetine containing a PSA peptide substrate have also been developed for prostate cancer treatment (Wu and Hu, 2016; Akinboye et al., 2017).

Caspases are proteases involved in programmed cell death. Among them, caspase-3 is closely related to apoptosis and is activated by both intrinsic and extrinsic pathways, making it a focus for cancer therapy (Chung et al., 2018). After tumors are treated with targeted therapy or radiotherapy, they express caspases, and caspase-responsive prodrugs can elicit cytotoxic effects to the related cancer cells. Byun and co-workers developed a caspase-cleavable prodrug, called EMC-DEVD-DOX, in which EMC (ε-maleimidocaproic acid) and DOX were connected through a DEVD peptide spacer (a widely used substrate of caspase-3). This small molecule prodrug can selectively target the irradiated local tumor and release free DOX with a substantially long plasma half-life (Chung et al., 2016). Likewise, MPD02, developed by their group, conjugates MMAE to the C-terminus of the KGDEVD peptide via a self-eliminating linkage and introduces EMC to the side chain of lysine (Chung et al., 2019). These caspase-activatable prodrugs could be used as an adjuvant of radiotherapy and other therapeutic methods to increase the therapeutic efficiency of cancer treatment.

In addition to the above mentioned, small molecule prodrugs that can be activated by other kinds of enzymes have also been developed (Zhang H. et al., 2018; Sun et al., 2019; Fukai et al., 2021; Rango et al., 2021; Liu et al., 2023; Tyagi et al., 2023). They are highly effective in targeting tumors and enhancing therapeutic efficacy.

In conclusion, enzyme-responsive small molecule prodrugs exploit the elevated or specific expression of certain enzymes in tumor cells to release active drug compounds selectively within the tumor microenvironment. These prodrugs remain inactive until they encounter the target enzyme, which catalyzes a chemical reaction to release the active drug. For instance, carboxylesterase-responsive prodrugs like gemcitabine and doxorubicin are activated by esterase enzymes, leading to targeted drug release and tumor inhibition. Similarly, β-galactosidase and β-glucuronidase-responsive prodrugs have shown promising results in various cancer models, using glycosidic and glucuronic acid residues to release cytotoxic agents specifically in tumor tissues. This strategy minimizes off-target effects and enhances therapeutic efficacy by ensuring that the active drug is released predominantly within the tumor. However, the heterogeneous expression of these enzymes in different tumors presents a challenge, necessitating further optimization and the development of highly specific enzyme-substrate pairs. Despite these challenges, enzyme-responsive prodrugs represent a powerful approach to improving the selectivity and effectiveness of cancer treatments.

### 2.2 GSH-responsive small molecule prodrugs

Glutathione (GSH) is a vital cellular metabolite that is essential for maintaining redox homeostasis in cells. Its role in regulating cellular health and disease is of utmost importance (Balendiran et al., 2004; Montero and Jassem, 2011; Dai et al., 2022; Shi et al., 2022). The intracellular GSH concentration of tumors (around 10 mM) is up to 40 times higher than that of healthy cells (Morales et al., 2005) and 100–1,000 times higher than the extracellular environment in normal cells (<10 μM) (Li et al., 2019). Consequently, various GSH-responsive small molecule prodrugs have been developed for cancer therapy, which can more rapidly release drugs in cancer cells in comparison with normal cells.

**FIGURE 6:**
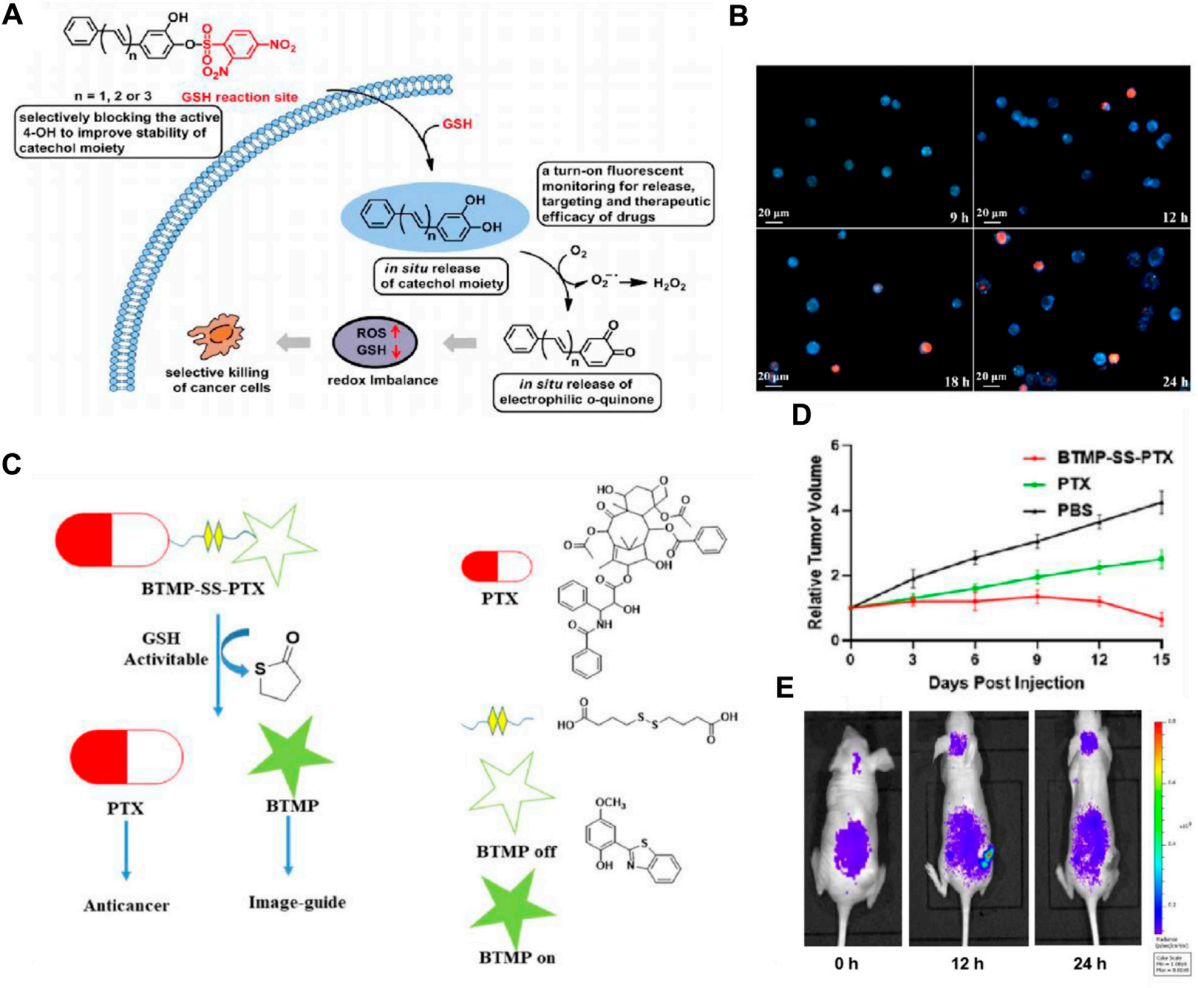
**(A)** Schematic depiction of designing glutathione-activated catechol-type diphenylpolyenes as small molecule anticancer prooxidative prodrugs. **(B)** Fluorescence imaging to assess the therapeutic efficacy of PDHH following its activation by GSH in SW620 cells. SW620 cells were treated with PDHH (15 μM) for 9–24 h, followed by PI staining. Adapted with modification from Ref. (Bao et al., 2019) ^©^ 2019 Elsevier. **(C)** Proposed method for traceable activation of BTMP-SS-PTX prodrug. **(D)** Comparison of tumor volume after treatment with PBS, BTMP-SS-PTX (equivalent to paclitaxel), and PTX (5 mg/kg). **(E)** Relative fluorescent ratio of tumors. Adapted with modification from Ref. (Ye et al., 2021) ^©^ 2021 Elsevier.

It is reported that 2,4-dinitrobenzenesulfonyl derivatives’ phenyl sulfide bond can be efficiently cleaved by GSH and serves as a cleavable site in the prodrug for drug release (Shibata et al., 2008; Wang et al., 2017). Based on this strategy, Fang’s group designed an anticancer small molecule prodrug that is triggered by GSH and releases the chemotherapeutic agent mechlorethamine. The biological studies indicated that the prodrug has good selectivity and growth inhibition on tumor cells (Xu et al., 2015). In addition, Zhou and colleagues developed three pro-catechol-type diphenylpolyenes as small molecule-based anticancer prodrugs with prooxidative properties. They protected the catechol moiety by converting 2,4-dinitrobenzenesulfonates (DNBS) to phenols through a GSH-mediated process (Bao et al., 2019). Particularly, the highly reactive 4-OH group on the catechol moiety was selectively blocked by DNBS. Their hypothesis was that the high GSH levels in cancer cells would activate the masked 4-OH group, releasing the catechol moiety locally within cancer cells, while remaining inactive in healthy cells. The released catechol moiety would then undergo *in situ* oxidation to *o*-quinone, generating reactive oxygen species, leading to selective cancer cell death (Figure 6A). The prodrug PDHH is superior to 5-FU and DOX, and its ability to kill SW620 cells (IC_50_ = 4.3 μM) is much greater than that of L02 cells (IC_50_ = 42.3 μM). Fluorescence imaging revealed that SW620 cells treated with 15 μM PDHH for 24 h displayed classic indicators of apoptosis, such as cytoplasmic shrinkage, the presence of vacuoles, and the formation of apoptotic bodies (Figure 6B).

Moreover, the disulfide bond also possesses relatively high reactivity with GSH and can be cleaved rapidly, thereby inducing the release of the targeted drug and the generation of fluorescent change (Wang et al., 2014). Numerous disulfide-containing prodrugs have been developed for cancer imaging and treatment (Wang et al., 2016; Botella et al., 2016; Kong et al., 2016). Zhu’s group designed and synthesized a novel small molecule anticancer prodrug, BTMP-SS-PTX, which combines 2-(benzo[d]thiazol-2-yl)-4-methoxyphenol (BTMP) with PTX through a disulfide bond linkage (Ye et al., 2021). When exposed to the elevated levels of GSH found in tumors, the prodrug undergoes activation and breaks down to release PTX and visible BTMP, allowing for the visualization and tracking of the free drug (Figure 6C). After the injection of the drug (100 μM) for 48 h, BTMP-SS-PTX exhibited lower toxicity to non-cancer cells 293T, and better anticancer activity in HepG2, MCF-7, and HeLa cells than free drugs. In the HeLa-xenograft mouse model, the prodrug significantly inhibited tumor growth (Figure 6D). *In vivo* imaging data demonstrated a gradual release of BTMP-SS-PTX at the tumor site, showcasing an increase in fluorescence intensity peaking at 12 h and significantly decreasing after 24 h (Figure 6E). In order to accomplish real-time monitoring of drug release during drug delivery, Zeng and colleagues developed theranostic GSH-responsive small molecule prodrugs based on fluorescence resonance energy transfer (FRET) (Hu and Zeng, 2017). In cancer cells exhibiting elevated GSH levels, the disulfide bonds are effectively broken, leading to the interruption of the FRET process. This results in a dual fluorescence response and the targeted release of CPT. Additionally, Brown’s group developed a theranostic CA-4 prodrug that releases the drug through the cleavage of the disulfide bond and contains a NIR fluorophore enabling timely monitoring of the cleavage (Kong et al., 2017). These strategies have offered new approaches for cancer diagnosis and treatment.

Generally, GSH-responsive small molecule prodrugs leverage the elevated levels of glutathione (GSH) found in tumor cells to achieve selective drug release. These prodrugs are designed with disulfide bonds or other GSH-reactive moieties that are cleaved in the presence of high GSH concentrations, which are typically 100–1,000 times higher in cancer cells compared to normal cells. This cleavage triggers the release of the active drug specifically within the tumor microenvironment. For instance, doxorubicin and paclitaxel prodrugs utilizing GSH-sensitive linkers have demonstrated enhanced targeting and reduced systemic toxicity in preclinical models. By exploiting the redox imbalance in cancer cells, GSH-responsive prodrugs provide a targeted delivery system that minimizes side effects and improves therapeutic outcomes. Despite the promising potential, challenges such as variability in GSH levels among different tumors and potential resistance mechanisms need to be addressed through further research and optimization.

### 2.3 ROS-responsive small molecule prodrugs

Malignant tumors exhibit elevated levels of ROS due to altered metabolism and signaling pathways (Trachootham et al., 2009; Liou and Storz, 2010). Key ROS include hydrogen peroxide (H₂O₂), superoxide anions (O₂⁻), and hydroxyl radicals. Among these, H₂O₂ is particularly important as it is stable and produced by nearly all sources of oxygen radicals (Lee and Shacter, 2000), this characteristic makes H₂O₂ a promising target for developing new ROS-responsive prodrugs with high selectivity for cancer cells (Lu et al., 2020).

The reaction between boronates and H_2_O_2_ is biocompatible and bioorthogonal. In the meantime, boronic acids and their esters apparently do not possess inherent toxicity, and the hydrolysis end product, boric acid, is deemed non-toxic to humans (Peng and Gandhi, 2012). Indeed, boronated H_2_O_2_-activatable small molecule prodrugs of SN38, DOX, 5-FU, and GEM have been devised for the selective cancer treatment and have demonstrated promising results *in vivo* (Wang et al., 2016; Ai et al., 2019; Matsushita et al., 2019; Skarbek et al., 2019). Moreover, considering DOX’s dose-dependent cardiotoxicity, which is partly attributed to its excessive production of ROS, Lukesh and team created a unique boronated hybrid codrug incorporating H_2_O_2_-responsive DOX, capable of releasing H_2_S through COS hydrolysis. This novel formulation aims to preserve DOX’s antitumor properties while minimizing its cardiotoxic effects (Hu et al., 2022).

**FIGURE 7:**
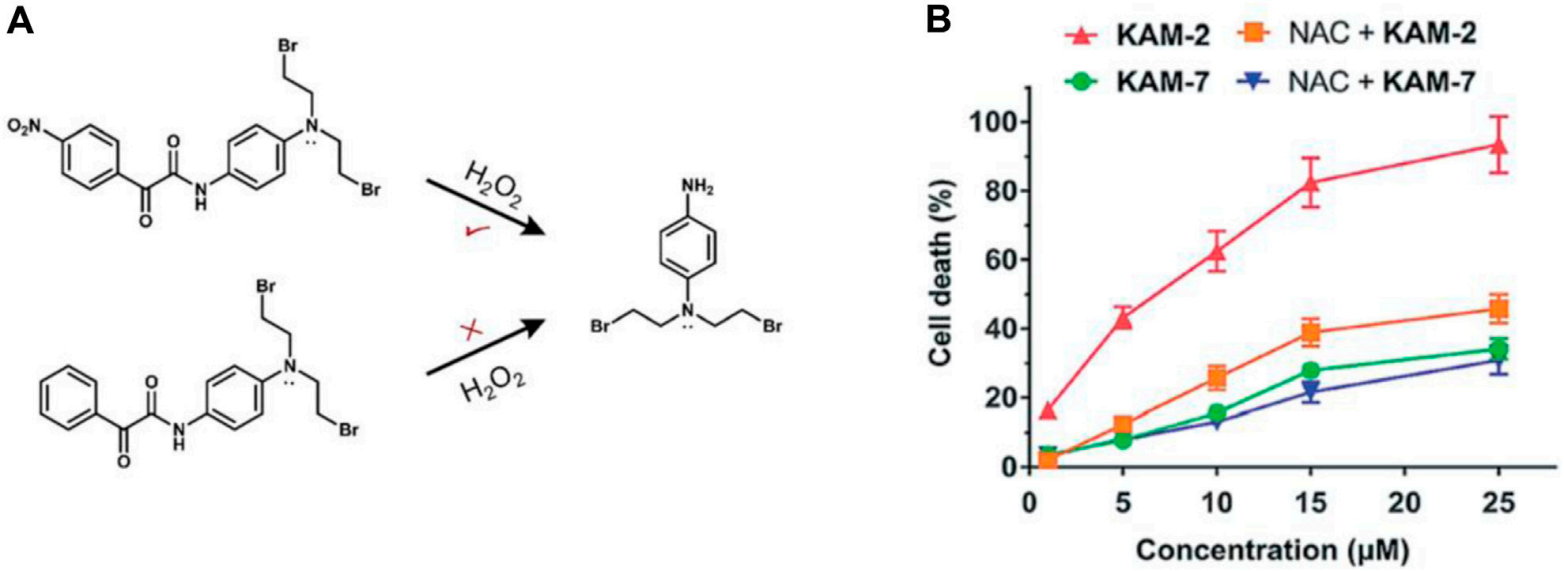
**(A)** Structures of prodrug KAM-2. **(B)** Assessing the impact of NAC on the cytotoxicity of KAM-2 and KAM-7 in HL-60 cells following a 72-h incubation period. Adapted with modification from Ref. (Meng et al., 2019) ^©^ 2019 Royal Society of Chemistry.

Yet, the clinical application of H_2_O_2_-responsive prodrugs utilizing phenylboronic acid/ester components is constrained by challenges including non-specific interactions between boronic acid and bio-diols, the susceptibility of boronic acid esters to hydrolysis, and the limited specificity of boronic acid esters for H_2_O_2_. Recently, a strategy based on an α-ketoamide structure to construct a novel H_2_O_2_-responsive nitrogen mustard prodrug KAM-2 has been reported by the Yin group (Figure 7A) (Meng et al., 2019).

The strong electron-withdrawing nitro group of KAM-2 increases the carbonyl group’s electrophilicity and enabling it to react with H_2_O_2_ significantly faster (Xie et al., 2016). Due to the significant role of the nitro group of KAM-2 in H₂O₂-induced nucleophilic attack, a non-nitro analog, KAM-7, which is resistant to H₂O₂, was synthesized as a negative control. KAM-2 showed stability without H₂O₂ and had low cytotoxicity against cancer cells. In the HL-60 cell line, KAM-2 demonstrated a strong antiproliferative effect, which was reduced by pretreatment with the H₂O₂ scavenger N-acetylcysteine (NAC). In contrast, KAM-7 was inert to H₂O₂ and showed low cytotoxicity toward HL-60 cells, and pretreatment with NAC did not diminish its toxicity (Figure 7B). The researchers subsequently illustrated the DNA-damaging and apoptosis-inducing effects associated with the liberated nitrogen mustard. This serves as a novel approach in the development of ROS-responsive small molecule prodrugs.

### 2.4 Bioreductive reactive small molecule prodrugs

Approximately 50% of cancer patients with solid tumors will receive platinum (Pt)-based anticancer drugs during chemotherapy (Miller et al., 2020). Pt (Ⅱ)-based treatments, such as oxaliplatin, carboplatin, and cisplatin, are approved for cancer therapy. However, their use is often limited by severe dose-dependent side effects, poor tumor specificity, and intrinsic or acquired cisplatin resistance (Kelland, 2007). To address these issues, researchers have developed Pt (Ⅳ)-based prodrugs.

Unlike the square-planar Pt (Ⅱ) complex, the octahedral Pt (Ⅳ) complex has two additional axial ligands, allowing adjustments to its biological properties like lipophilicity, selectivity, and redox stability. The Pt (Ⅳ) complex can be reduced to Pt (Ⅱ) species and release axial ligands in the presence of biological reducing agents such as ascorbic acid (AsA) and GSH (Johnstone et al., 2016). GSH is consumed during Pt (Ⅳ) complex activation, which helps overcome cisplatin resistance and reduces side effects (Galluzzi et al., 2012). As a result, Pt (Ⅳ) complexes are being explored as the next generation of platinum-based anticancer drugs. Gou’s group designed Pt (Ⅳ)-based prodrugs with an indomethacin moiety linked to biotin for targeting tumors (Hu et al., 2017), while Xu’s group attempted to conjugate riluzole into Pt (Ⅳ)-based prodrugs to enhance anticancer activity and reduce side effects, achieving promising results (Li et al., 2023).

**FIGURE 8:**
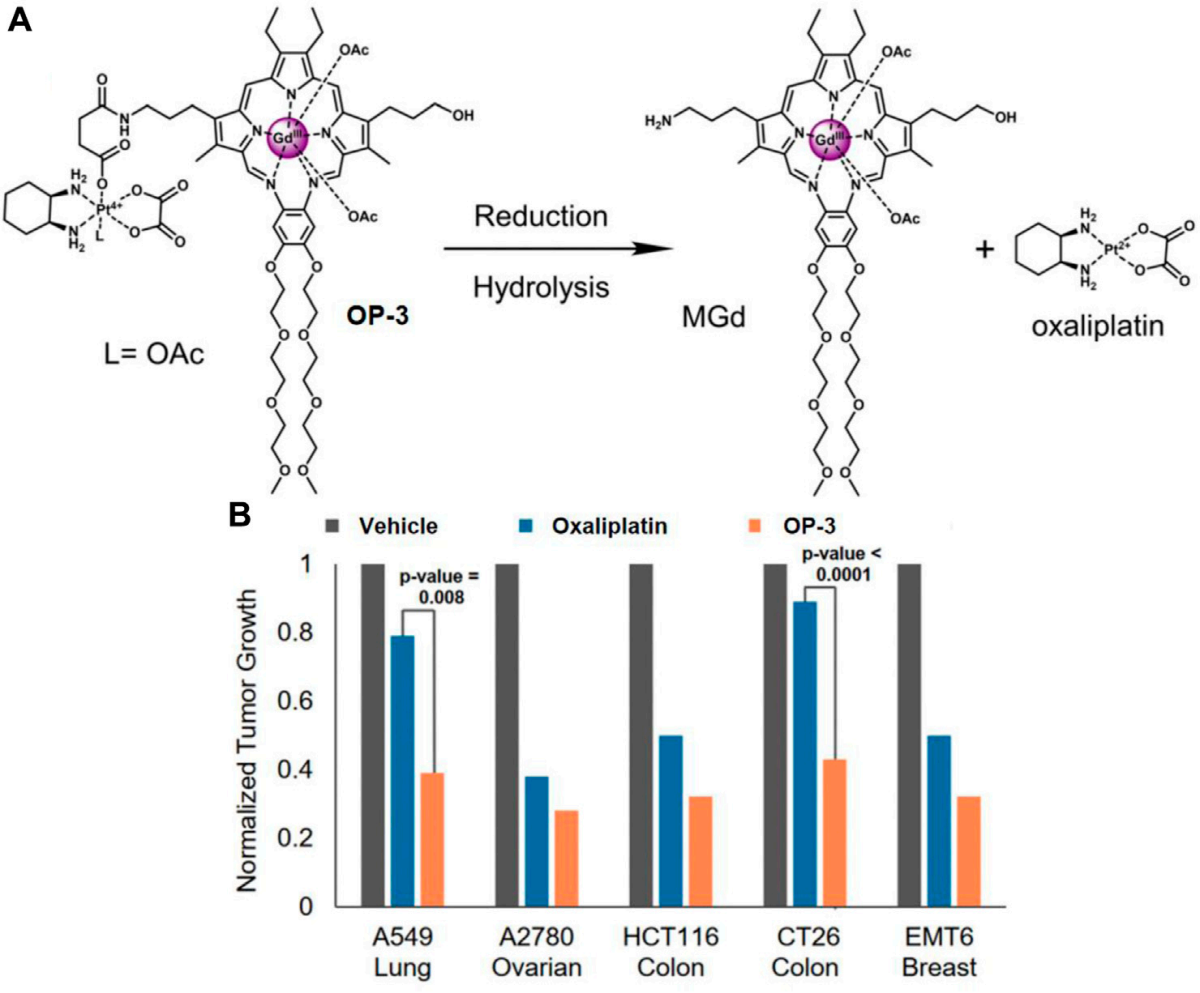
**(A)** Chemical structure of OP-3 and the release of therapeutic MGd and oxaliplatin through hydrolysis and reduction. **(B)** *In vivo* efficacy of oxaliplatin (4 mg/kg) and OP-3 (70 mg/kg) in HCT116, A2780, and A549 tumor-bearing mice, and syngeneic tumor models (EMT6, CT26). Adapted with modification from Ref. (Thiabaud et al., 2020) ^©^ 2020 National Academy of Sciences.

Motexafin gadolinium (MGd) is a type of expanded porphyrin used as a redox mediator. Recent studies confirm that MGd can catalyze the intracellular reduction of oxaliplatin-based Pt (Ⅳ) prodrugs to cytotoxic Pt (Ⅱ) (Thiabaud et al., 2016). Sessier and colleagues reported a Pt (Ⅳ)-based texaphyrin conjugate OP-3 (Thiabaud et al., 2020). The conjugate consisted of MGd, linked to the oxaliplatin Pt (Ⅳ) derivative via a succinate linker, with an acetoxy group as the axial ligand (Figure 8A). OP-3 demonstrated good stability in serum, and the reduction of Pt (Ⅳ) was confirmed using reverse-phase high-performance liquid chromatography (HPLC). *In vitro* experiments revealed that OP-3 had broad antiproliferative effects compared to oxaliplatin across multiple cell lines, including those resistant to platinum. In mice with xenografts of A549, A2780, HCT116, CT26, and MET6, OP-3 (70 mg/kg per dose on days 1–13) significantly reduced tumor volume compared to the vehicle and oxaliplatin (4 mg/kg per dose on days 1–13) (Figure 8B). These findings suggest that OP-3 is a more effective candidate for slowing and inhibiting tumor growth, offering a promising approach for the clinical translation of Pt (Ⅳ)-based prodrugs.

### 2.5 Hypoxia-activated small molecule prodrugs

Owing to insufficient blood circulation in tumor tissues, hypoxia is a common feature in solid tumors. The oxygen supply fails to meet the metabolic requirements of tumor cells, which subsequently aggravates hypoxia along with tumor growth, resulting in the intratumoral microenvironment presenting a notable hypoxic phenotype (Emami Nejad et al., 2021). Nevertheless, hypoxia presents a promising target for cancer diagnosis and treatment. It is well recognized that reducing enzymes such as azoreductase and nitroreductase are overexpressed in hypoxic solid tumors. These enzymes can respectively reduce azoaromatic and nitro-aromatic derivatives. As a result, novel hypoxia-activated small molecule prodrugs incorporating azoaromatic and nitroaromatics units as hypoxia-responsive protective groups have been created (Verwilst et al., 2017; Kim et al., 2018; Peng et al., 2019).

**FIGURE 9:**
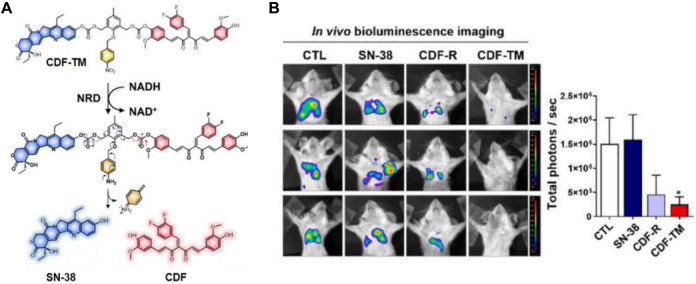
**(A)** Chemical structure and activation mechanism of CDF-TM. **(B)** Representative BLI of metastases in control (DMSO), CDF-R (0.001 mmol/kg), CDF-TM (0.001 mmol/kg), and SN-38 (0.001 mmol/kg) treated mice. Adapted with modification from Ref. (Kim et al., 2022) ^©^ 2022 Elsevier.

Cancer stem-like cells (CSCs) pose a challenge to the effective treatment of triple-negative breast cancer (TNBC) and contribute to the development of chemoresistance, leading to poor prognosis. To address this, Kim’s group developed a small-molecule-based binary prodrug, CDF-TM, which is selectively activated in hypoxic environments to release the anticancer drug SN-38 and the CSC-suppressing agent 3,4-difluorobenzylidene curcumin (CDF) (Kim et al., 2022). CDF-TM consists of a self-immolative linker and a hypoxia-responsive linker, nitrobenzene, to connect SN-38 and CDF (Figure 9A). The reference compound, CDF-R, was similarly designed by attaching only CDF to the linker. In BALB/c female mice with breast cancer stem cell (BCSC)-enriched 4T1 tumors, CDF-TM significantly inhibited tumor growth. Bioluminescence imaging showed that both control and SN-38-treated groups had a higher presence of spreading BCSCs in the lungs, whereas fewer spreading cells were observed in the CDF-R and CDF-TM treated groups, with only a weak signal in the CDF-TM group (Figure 9B). This novel small molecule prodrug offered a distinct therapeutic option for TNBC.

In addition, the incorporation of nitroimidazole groups has also been observed in activable chemotherapeutics. Kowol’s group developed a hypoxia-activatable 2-nitroimidazole-based prodrug (Bielec et al., 2020). Anaplastic lymphoma kinase (ALK) and mesenchymal-epithelial transition factor (c-MET) are pivotal receptor tyrosine kinases involved in tumor proliferation (Holla et al., 2017; Zhang et al., 2018). Under hypoxic conditions, the prodrug displayed the liberation of the ALK and c-MET inhibitor crizotinib. Notably, the prodrug exhibited excellent stability in serum and effectively suppressed c-MET phosphorylation and tumor cell proliferation *in vivo*.

### 2.6 Acidic pH-responsive small molecule prodrugs

Due to the high glycolytic metabolism of cancer cells, their microenvironment tends to be acidic (pH 6.5–7.2), a key feature of cancers (Piasentin et al., 2020). The acidic environment in solid tumor tissues is crucial for cancer cell proliferation, migration, invasion, metastasis, drug resistance, and immune evasion (Boedtkjer and Pedersen, 2020). Based on this characteristic of cancers, a large number of acid-responsive prodrug systems have been reported (Hou et al., 2017; Teng et al., 2022). The use of acid-sensitive breakable hydrazones is a common strategy (Jin et al., 2023).

**FIGURE 10:**
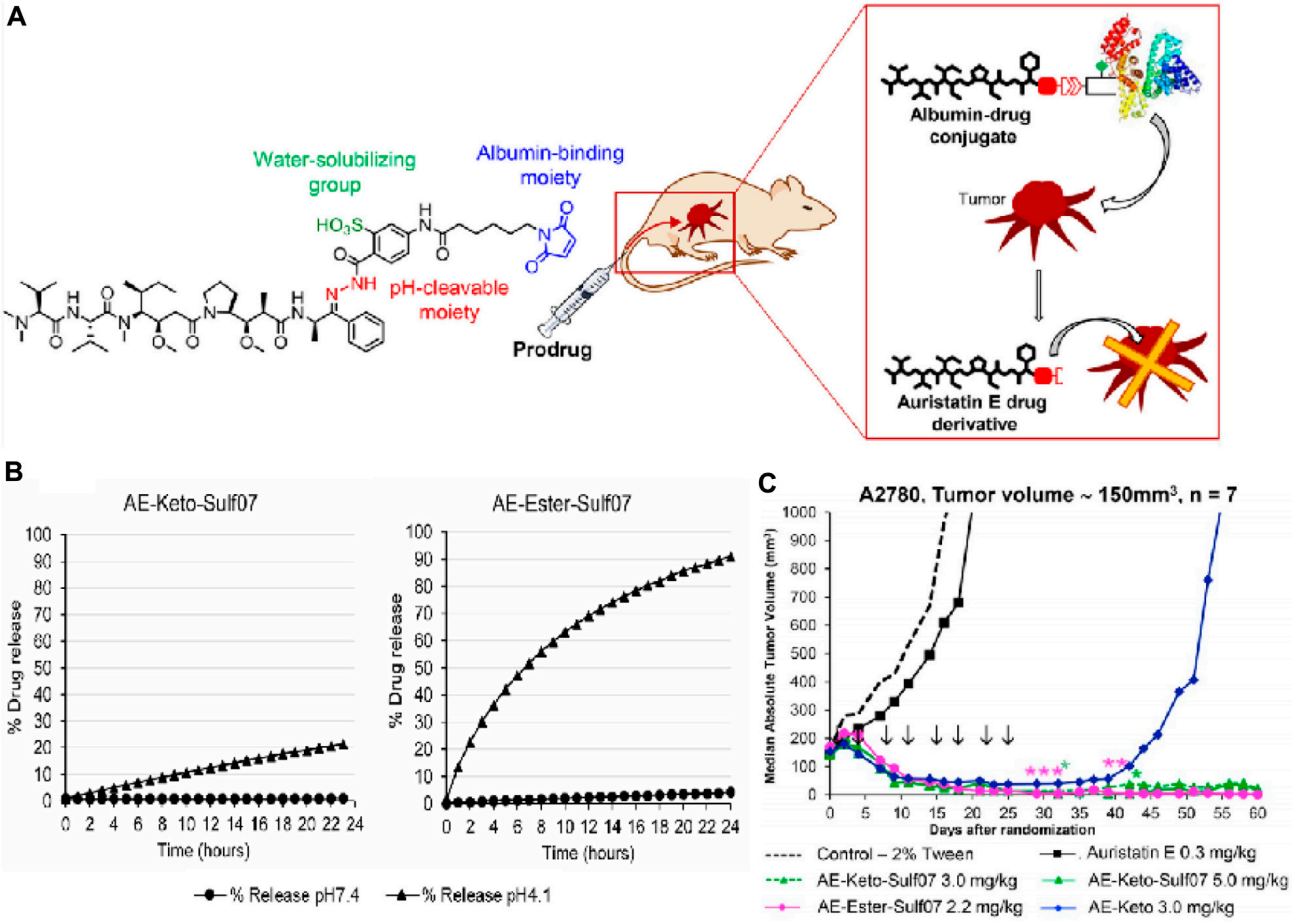
**(A)** Chemical structures of prodrug AE-Keto-Sulf07. **(B)** Release of AE-Keto and AE-Ester from AE-Keto-Sulf07 and AE-Ester-Sulf07 at pH 7.4 and pH 4.1. **(C)** Comparison of tumor growth curves for AE-Keto, AE-Ester-Sulf07, and AE-Keto-Sulf07 against AE and control groups in human tumor xenograft models using nude mice. Adapted with modification from Ref. (Pes et al., 2019) ^©^ 2019 Elsevier.

Kratz and his colleagues reported two acid-responsive albumin-binding prodrugs, AE-Ester-Sulf07 and AE-Keto-Sulf07, for the targeted delivery of the cytotoxic tubulin-disrupting peptide auristatin E to cancer cells (Pes et al., 2019). In this theranostic, the albumin-binding moiety was linked to AE-Ester and AE-Keto (two carbonyl-containing derivatives of auristatin E) via an optimized maleimide-bearing hydrazide linker (Figure 10A). Using LC-MS, it was discovered that 91%–99% of both prodrugs were bound to albumin within 2 min after incubation in murine and human plasma. HPLC analysis confirmed that the stability of the prodrugs under simulated physiological conditions (pH 7.4) was significantly higher compared to the free drug. In acidic conditions (pH 4.1), 21% of AE-Keto was released after 24 h, while AE-Ester-Sulf07 released 90% AE-Ester after 24 h (Figure 10B). In nude mice with human tumor xenografts, both albumin-binding prodrugs demonstrated remarkable anticancer effects compared to the parent drug auristatin E (Figure 10C). All in all, AE-Ester-Sulf07 and AE-Keto-Sulf07 are innovative albumin-binding prodrugs with great potential for the delivery of auristatins toward solid tumors.

Zhang and colleagues demonstrated another hydrazone-based prodrug of DOX for evaluation in U87 cells. This prodrug consists of DOX, a coumarin fluorophore, and an αvβ3 integrin targeting component (Li et al., 2014). Utilizing a hydrazone maleimide linker, DOX was linked to the structure, causing quenching of the coumarin fluorescence by DOX molecules in the prodrug. Consequently, the prodrug exhibited diminished fluorescence intensity at pH 7.4. Upon lowering the pH to 5.0, a swift increase in fluorescence emission at 330 nm was observed, indicative of hydrazone unit hydrolysis and subsequent DOX release. This feature enabled real-time tracking of DOX release in *in vitro* settings.

### 2.7 Light-responsive small molecule prodrugs

Due to the exceptional advantages of optical signal control, including non-invasiveness, high spatial resolution, and precise spatio-temporal control, numerous light-activated compounds have been synthesized for use in chemotherapy (Dcona et al., 2020; Vickerman et al., 2021). Early strategies for light-responsive applications utilized nitrobenzyl photoresponsive groups (Mo et al., 2016). However, activation of this unit requires shorter excitation wavelengths (below 500 nm), which often result in limited tissue penetration and possible phototoxicity (Hou et al., 2018). Recently, research has concentrated on developing light-activatable systems that operate with longer excitation wavelengths and demonstrate enhanced photoconversion efficiencies.

Photodynamic therapy (PDT) employs photosensitizers (PS) that are activated by visible to near-infrared light to produce singlet oxygen (^1^O_2_), leading to cellular toxicity. However, ^1^O_2_ has a brief lifespan and limited diffusion range, restricting its damage within the cell diameter. This confinement means there is no bystander effect, often resulting in suboptimal therapeutic outcomes for PDT (Arya et al., 2012). You and colleagues have innovated by creating a light-activated prodrug, Pc-(L-CA4)_2_, which consists of a fluorescent phthalocyanine PS, the anticancer drug CA-4, and a ^1^O_2_-sensitive linker (Rajaputra et al., 2016). Research demonstrated that upon irradiation with a 690 nm laser, the prodrug generates ^1^O_2_, causing localized tumor damage while simultaneously releasing CA-4. This release of CA-4 leads to more extensive and prolonged damage, effectively killing tumor cells that survive the initial PDT. Thus, the prodrug Pc-(L-CA4)_2_ effectively surpasses the spatial and temporal constraints of ^1^O_2_ in PDT, significantly enhancing its antitumor efficacy.

**FIGURE 11:**
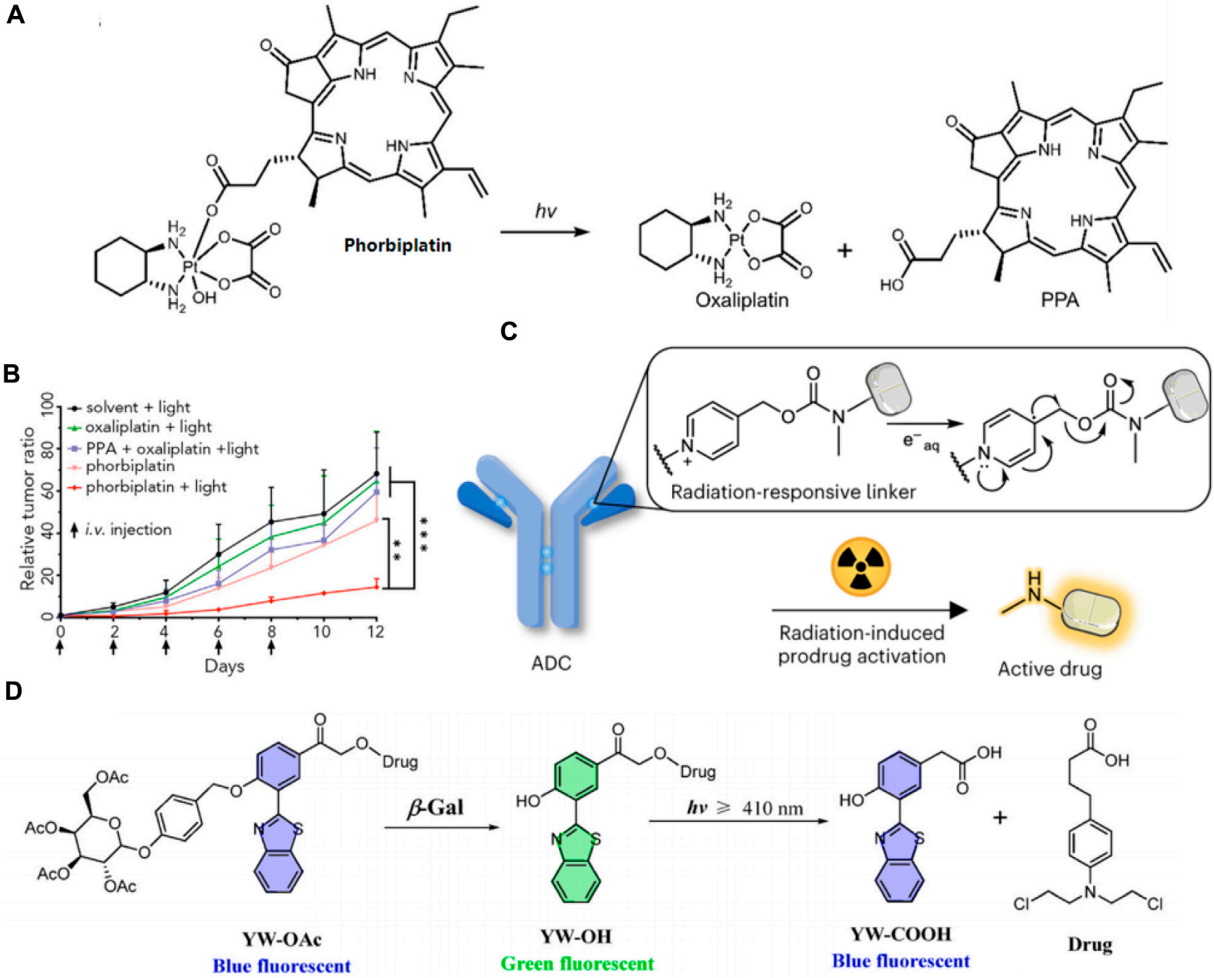
**(A)** The molecular structure of phorbiplatin and the mechanism of oxaliplatin release triggered by light at 650/660 nm. **(B)** Time course of tumor growth suppression compared to controls. Adapted with modification from Ref. (Wang et al., 2019) ^©^ 2019 Elsevier. **(C)** Schematic representation of a radiation-responsive ADC. Adapted with modification from Ref. (Fu et al., 2024) ^©^ 2024 Springer Nature. **(D)** Diagram of the YW-OAc prodrug fluorescent probe. Adapted with modification from Ref. (Chen et al., 2023) ^©^ 2023 Elsevier.

Zhu’s group recently synthesized a light-activatable oxaliplatin Pt (Ⅳ) conjugate PS, named phorbiplatin, which operates at wavelengths of 650/660 nm. This photosensitizer utilizes pyropheophorbide α (PPA), known for its high absorbance at 650 nm and efficient generation of ^1^O_2_. The ^1^O_2_-producing PPA was attached to the axial position of the oxaliplatin Pt (Ⅳ) prodrug (Figure 11A) (Wang et al., 2019). Upon exposure to low-intensity red light at 650 nm, the Pt (Ⅳ) is converted to Pt (Ⅱ) and PPA is simultaneously released. Phorbiplatin demonstrated enhanced cytotoxic effects on cancer cells when activated by light compared to dark conditions. In experiments using the 4T1 tumor-bearing mouse model, phorbiplatin treated with light (660 nm, 100 mW cm^−2^, 10 min) exhibited superior antitumor effects compared to both standard oxaliplatin and a combination of oxaliplatin with PPA (Figure 11B). This is the first study that uses red light to control the activation of platinum prodrugs *in vivo*, and this work is expected to motivate other researchers to develop novel light-responsive Pt (Ⅳ) prodrugs.

Fu and colleges have developed a novel approach to reduce systemic toxicity in cancer therapy by using radiotherapy-induced prodrug activation (Fu et al., 2024). Researchers identified N-alkyl-4-picolinium (NAP) as an efficient caging group that releases active molecules upon exposure to radiation. The study developed a NAP-derived carbamate linker that, when integrated into antibody-drug conjugates (ADCs), released fluorophores and toxins upon irradiation (Figure 11C). These ADCs were effective in living cells and tumor-bearing mice, demonstrating a marked antitumor effect. The research highlighted the potential of using radiation-removable protecting groups for creating next-generation ADCs with enhanced stability and therapeutic efficacy. This method showed promise for targeted drug activation, minimizing damage to healthy tissues and improving the overall effectiveness of cancer treatments.

Furthermore, in order to achieve real-time visualization of ovarian cancer cells during chemotherapy, Hou and his colleagues devised a novel prodrug fluorescent probe, YW-OAc, which is capable of tracing cancer cells while delivering chemotherapeutic agents (Chen S. et al., 2023). YW-OAc is composed of three key elements: D-acetyl-galactose residue, 2-(2′-hydroxyphenyl) benzothiazole as a fluorescent marker, and the anticancer agent chlorambucil. Its fluorescence shifts from blue to green after reacting with β-Gal in cancer cells. Following light exposure (≥410 nm), the green fluorescence reverts to blue upon chlorambucil’s release (Figure 11D). In normal cells, the fluorophore remains unchanged, irrespective of light exposure. Conversely, in OVCAR-3 cancer cells that overexpress β-Gal, the glycosidic bond in YW-OAc is cleaved, releasing the fluorophore, which is then subject to photolysis by irradiation, thus liberating the drug. YW-OAc exhibits low toxicity to normal cells regardless of irradiation and shows no significant dark toxicity to ovarian cancer cells but demonstrates marked phototoxicity. YW-OAc holds promise as an effective bioimaging and diagnostic tool.

### 2.8 Bioorthogonal reaction-based small molecule prodrugs

Bioorthogonal reaction-based small molecule prodrugs represent a novel approach in the development of targeted cancer therapies. These prodrugs are designed to undergo specific chemical reactions that are orthogonal, or non-interfering, with biological processes. The unique aspect of bioorthogonal reactions is that they can occur in living systems without disrupting native biochemical pathways, ensuring high selectivity and minimizing off-target effects. Typically, these reactions involve the conversion of an inactive prodrug into an active drug through a catalyst or a trigger present within the tumor microenvironment. This strategy enables precise drug activation at the tumor site, enhancing therapeutic efficacy while reducing systemic toxicity. Recent advancements have demonstrated the potential of bioorthogonal prodrugs in achieving targeted and controlled drug release, paving the way for more effective and safer cancer treatments.

**FIGURE 12:**
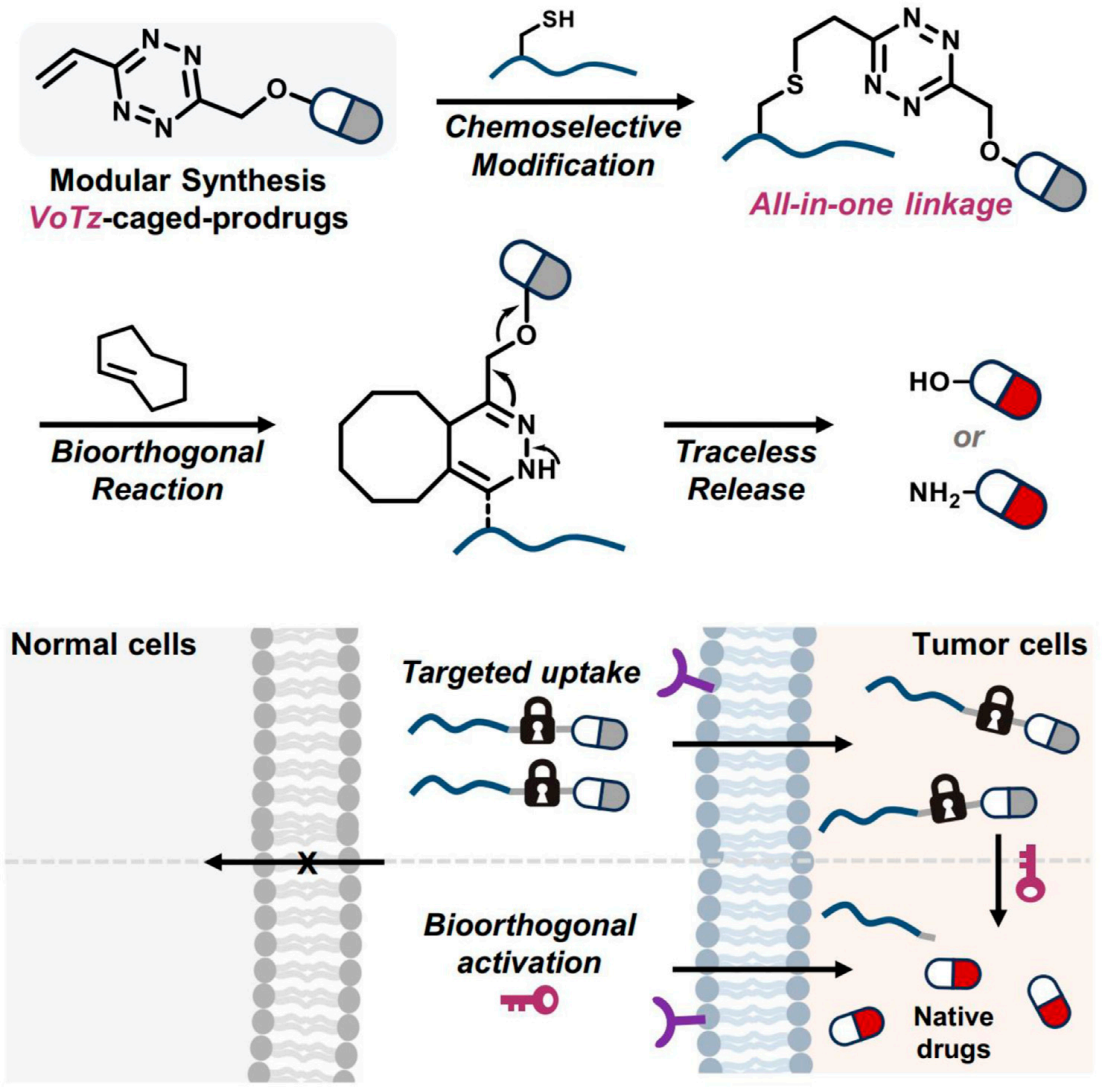
The development of bioorthogonal activable peptide-drug conjugates (PDCs) involves the creation of an all-in-one tetrazine reagent that facilitates chemoselective labeling and prodrug synthesis. Adapted with modification from Ref. (He et al., 2024) ^©^ 2024 Springer Nature.

In He and coworkers’ study, 3-vinyl-6-oxymethyl-tetrazine (voTz) was introduced as a versatile reagent for the modular preparation of bioorthogonal activable prodrugs (He et al., 2024). The voTz reagent allowed for cysteine-selective labeling and the creation of peptide-drug conjugates (PDCs) with high stability and specificity (Figure 12). These PDCs remained inactive in the bloodstream and became activated only upon reaching the tumor site, where bioorthogonal cleavage reactions triggered drug release. This design significantly enhanced the targeting efficiency and therapeutic potency of the prodrugs while minimizing off-target toxicity. *In vivo* studies demonstrated the therapeutic efficacy and safety of this approach, suggesting that voTz-based prodrugs could be a promising strategy for precision cancer therapy. The broad applicability of functional groups and the chemoselective modification capability of voTz highlight its potential for developing next-generation biopharmaceuticals and biomaterials.

## 3 Comparison of small molecule prodrugs with conventional chemotherapeutics

### 3.1 Targeted delivery and activation mechanisms

Small molecule prodrugs are designed to remain inactive until they reach the tumor site, where specific enzymes, pH levels, or other biomarkers trigger their activation. This targeted delivery minimizes off-target effects and enhances drug concentration at the tumor site. In contrast, conventional drugs are active throughout the body, often leading to higher systemic toxicity. For example, enzyme-responsive prodrugs such as β-galactosidase and β-glucuronidase-activated prodrugs show lower toxicity in non-cancerous cells compared to traditional chemotherapeutics. This targeted activation allows for higher efficacy in killing tumor cells while reducing harmful side effects commonly seen with conventional chemotherapies (Senter and Sievers, 2012).

### 3.2 Reduced systemic toxicity and enhanced stability

By targeting the tumor microenvironment specifically, small molecule prodrugs reduce the risk of damaging healthy tissues. Prodrugs can be chemically modified to improve their solubility and stability, which are critical factors for drug delivery and efficacy. Conventional drugs may lack these modifications, leading to challenges in formulation and administration. Studies have shown that small molecule prodrugs can achieve better therapeutic outcomes by ensuring higher drug concentrations at the tumor site and reducing adverse effects. For instance, the GSH-responsive prodrug BTMP-SS-PTX demonstrated superior anticancer activity and lower toxicity compared to paclitaxel alone. This improvement in stability and solubility ensures that the drug remains effective during its transit through the body, ultimately leading to better patient outcomes (Haag and Kratz, 2006).

## 4 Conclusion

Prodrug strategies, crucial in cancer therapy, involve regulating the toxicity of anticancer drugs and have been adopted to create a variety of therapeutic agents for treatment, targeting, diagnosis, and imaging purposes. Such strategies enhance the allure of prodrug design as a pivotal method in oncological drug development. In this review, we classify these strategies according to their activation mechanisms. By directly modifying drugs that have already gained clinical approval, this approach substantially cuts down the time required for drug development, offering a route to enhance drug effectiveness and mitigate side effects. Although this approach shows promise for cancer chemotherapy, several challenges must still be overcome to design more tailored and effective prodrugs.

One major challenge is enzymatic heterogeneity within the tumor microenvironment, which can affect prodrug activation efficiency. Enzyme-responsive prodrugs require meticulous optimization of enzymes and their substrates. Although the linkers’ chemical bonds are generally less susceptible to physiological conditions, thus lowering the risk of non-specific prodrug activation, the possibility of premature or failed activation at incorrect sites remains. Many enzymes that are overexpressed in cancerous tissues are also found in normal tissues, necessitating the development of highly specific enzyme-substrate pairs and the exploration of multifunctional linkers that can respond to multiple tumor-specific stimuli.

Prodrug activation specificity is another significant challenge. Ensuring that prodrugs are activated only at the tumor site without affecting healthy tissues is critical for minimizing side effects. Strategies such as incorporating dual or multi-activatable systems that respond to multiple tumor-specific triggers can enhance activation specificity and therapeutic efficacy.

Additionally, the pharmacokinetics and pharmacodynamics (DMPK) of prodrugs must be finely tuned to reduce off-target effects and enhance therapeutic efficacy. This involves optimizing the stability, solubility, and distribution of prodrugs in the body. Novel delivery systems, such as nanoparticles and liposomes, can improve the bioavailability and tumor targeting of prodrugs (Chenguang et al., 2020; Kankala et al., 2021), thereby overcoming some of the DMPK challenges.

Looking ahead, the development of dual or multi-activatable prodrugs seems to be a leading strategy for achieving enhanced tumor specificity in targeted cancer treatments, as demonstrated in previous studies. If these prodrugs incorporate additional therapeutic modalities, such as photodynamic therapy, photothermal therapy, and immunotherapy, more effective cancer treatments may emerge, offering advancements over conventional chemotherapy. We anticipate that ongoing research will soon yield novel, activatable small molecule prodrugs that are clinically effective for cancer treatment.
